# Pneumonia prevention effects of perioperative oral management in approximately 25,000 patients following cancer surgery

**DOI:** 10.1002/cre2.264

**Published:** 2019-12-17

**Authors:** Yasuhiro Kurasawa, Yutaka Maruoka, Hideki Sekiya, Akihide Negishi, Hitoshi Mukohyama, Shiro Shigematsu, Jumpei Sugizaki, Kazunari Karakida, Masaru Ohashi, Masayuki Ueno, Yukihiro Michiwaki

**Affiliations:** ^1^ Division of Maxillofacial Surgery Graduate School, Tokyo Medical and Dental University Tokyo Japan; ^2^ Department of Oral Surgery Japanese Red Cross Musashino Hospital Musashino Japan; ^3^ Division of Oral and Maxillofacial Surgery Center Hospital of National Center for Global Health and Medicine Tokyo Japan; ^4^ Division of Oral Surgery, Graduate School Tokyo Medical and Dental University Tokyo Japan; ^5^ Department of Oral Surgery Toho University Omori Medical Center Tokyo Japan; ^6^ Department of Oral and Maxillofacial Surgery National Hospital Organization Yokohama Medical Center Yokohama Japan; ^7^ Department of Oral Surgery Yokohama City Minato Red Cross Hospital Yokohama Japan; ^8^ Department of Dentistry and Oral Surgery Tokyo Metropolitan Tama Medical Center Tokyo Japan; ^9^ Department of Dentistry Toranomon Hospital Tokyo Japan; ^10^ Department of Dentistry and Oral Surgery Tokai University Hachioji Hospital Hachioji Japan; ^11^ Department of Oral Surgery JCHO Tokyo Takanawa Hospital Tokyo Japan; ^12^ Division of Oral Health Sciences, Department of Health Sciences School of Health and Social Services Saitama Prefectural University Koshigaya Japan

**Keywords:** oral health, perioperative care, pneumonia

## Abstract

**Aim:**

We conducted a multicenter study to explore the risk factors of developing pneumonia and the effectiveness of perioperative oral management (POM) for the prevention of pneumonia in postsurgical patients.

**Methods and results:**

A survey covering eight regional hospitals was conducted over 4 years, from April 2010 to March 2014. Using the Diagnosis Procedure Combination database, a target group of 25,554 patients with cancer who underwent surgery was selected and assessed from a population of 346,563 patients without pneumonia on admission (sample population). The study compared the incidence of pneumonia and attempted to identify the significant predictive factors for its occurrence in these patients using multiple logistic regression analysis. Comparative assessment for the occurrence of pneumonia before and after POM implementation showed a significant incidence decrease after POM introduction in the target group, with no such change observed in the sample population. Multiple logistic regression analysis showed that the odds ratio for pneumonia occurrence after POM introduction was 0.44, indicating a reduced risk of pneumonia.

**Conclusion:**

POM in cancer patients was indeed effective in reducing the incidence of pneumonia in hospitals and thereby helped in preventing pneumonia during hospitalization.

## 1 INTRODUCTION

Pneumonia is a common and sometimes fatal complication following major cancer surgery. Previous studies have shown significant associations of postoperative pneumonia with both prolonged length of hospitalization and with mortality.(Juthani‐Mehta, De Rekeneire, Allore, et al., 2013; Khuri, Henderson, DePalma, et al., 2005; PROVE Network Investigators for the Clinical Trial Network of the European Society of Anaesthesiology et al., 2014)

Perioperative oral management (POM) was introduced into the Japanese universal health insurance system in April 2012 to prevent postoperative complications such as pneumonia in cancer patients. POM refers to professional oral care provided by dentists or dental hygienists and involves pretreatment evaluation of oral hygiene and administration of appropriate oral care and dental treatment in cases of high risk of infection. The goal of POM is to ensure good oral hygiene prior to initiating further treatment, as well as posthospitalization. Several studies have shown that perioperative oral care may help prevent complications such as pneumonia and surgical site infection after cancer surgery.(Hong, Gueiros, Fulton, et al., 2019; Ishimaru et al., 2018; Nishikawa et al., 2019; Sato et al., 2011; Soutome et al., 2017) However, as these were small‐scale studies and high‐evidence‐level studies are limited, it remains unclear whether POM can reduce pneumonia incidence.(Aoyama & Tamagawa, 2019)

The number of cancer patients is increasing; in 2016, there were 17.2 million cancer cases worldwide with an increase of 28% between 2006 and 2016.(Global Burden of Disease Cancer C et al., 2018) Therefore, the number of patients hospitalized for treatment is also expected to increase. However, the number of the patients that can be treated by dentists or dental hygienists is limited and it is difficult to perform POM for all cancer patients admitted to the hospital. It is therefore necessary to consider the factors that are implicated in the onset of pneumonia and to prioritize POM for patients who are at high risk. So far, the factors that expose hospitalized patients to increased risk of developing pneumonia remain unclear. For example, no study to date has compared the incidence of pneumonia in hospitalized patients by cancer type.

Here, we planned a multicenter collaborative study on the incidence of pneumonia in cancer patients and attempted to determine which cancer patients are at increased risk of developing pneumonia.

We screened these patients for cancer types with high risk of developing pneumonia during hospitalization and compared the incidence of pneumonia before and after the introduction of POM.

POM is only provided to cancer, organ transplantation, and cardiovascular surgery patients in the Japanese universal health insurance system. Our study only targeted cancer patients who underwent surgery, and we examined how often these patients developed pneumonia, whether there were any cancers associated with the development of pneumonia other than esophageal and lung cancers, which have already been reported,(Agostini et al., 2010; Ando, Ozawa, Kitagawa, Shinozawa, & Kitajima, 2000) and whether the incidence of pneumonia would be reduced by the initiation of POM.

The purposes of this study were to evaluate the effect of POM by comparing the preintroduction and postintroduction of POM periods, to statistically calculate the incidence and risk of pneumonia by cancer type and to determine base data for triaging hospitalized patients who undergo POM.

## 2 METHODOLOGY

This was a multicenter, collaborative, retrospective study using data from the Diagnosis Procedure Combination (DPC) database. The details of the DPC database have been described elsewhere.(Fuji, Akagi, Abe, et al., 2017; Nakamura, 2016; Okamoto et al., 2018; Okamoto, Uchiyama, Takemura, et al., 2013) The DPC data of hospitalized patients included all electronic records pertaining to clinical and medical care information.

Of all patients admitted to the participating institutions, only cancer patients who underwent surgery were selected using the DPC database.

### 2.1 Variables

The variables retrieved from the DPC database were sex, age, date of hospitalization, three categories of the diseases names (encompassing diseases that led to hospitalization, comorbid diseases at the time of hospitalization, and diseases that developed during hospitalization), and DPC codes.

### 2.2 Patients

Patients were recruited from eight regional central hospitals. All institutions adopted the bundled payment healthcare fee system using the DPC system.

Since POM was introduced into the Japanese universal health insurance system in April 2012, the period of analysis covered 2 years before (April 2010–March 2012) and after (April 2012–March 2014) its introduction. The sample population included 346,563 subjects, all of whom were medical patients hospitalized in any of the eight facilities during the 4‐year study period, excluding those who had pneumonia at the time of admission. All cancer patients who underwent surgery, excluding those who had pneumonia at the time of admission, were extracted from the sample population as the target group, ultimately including 25,554 patients.

### 2.3 Patient selection and data collection

The DPC database includes three categories of diseases, encompassing diseases that led to hospitalization, comorbid diseases at the time of hospitalization, and diseases that developed during hospitalization. For example, when a patient with hypertension is hospitalized for the purpose of treatment of stomach cancer, the disease that led to hospitalization would be registered as stomach cancer, and the comorbid disease at the time of hospitalization would be registered as hypertension. If this patient developed pneumonia during hospitalization, pneumonia would additionally be registered in the “disease that developed during hospitalization” field.

In this study, patients with no record of any type of pneumonia in the “disease that led to hospitalization” and “comorbid disease at the time of hospitalization” fields, but with record of pneumonia, irrespective of disease type, listed as a “disease that developed during hospitalization” were considered patients who developed pneumonia during hospitalization.

The patients that did not develop pneumonia during hospitalization included those with no description of pneumonia, irrespective of type, in any of the three fields described above. “Excluded cases” referred to patients with pneumonia, irrespective of type, listed in the “disease that led to hospitalization” and “comorbid disease at the time of hospitalization” fields.

It was difficult to determine using the DPC database the exact point in time when pneumonia occurred after admission; therefore, we designated any incident case of pneumonia as pneumonia during hospitalization, instead of using terms such as postoperative pneumonia.

**Figure 1:**
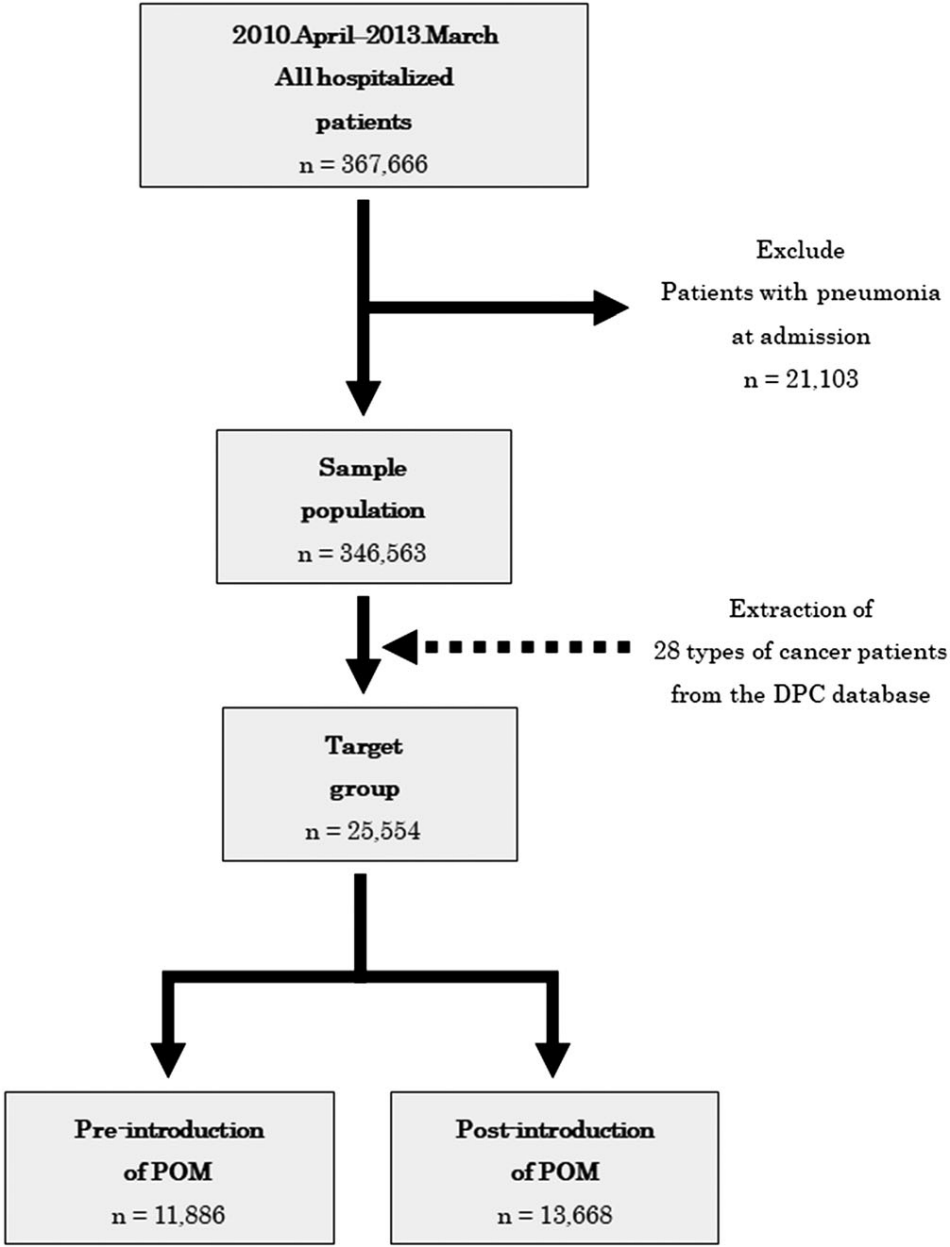
Scheme of patient selection

The DPC database includes a 14‐digit code called the DPC code. This code is specific to each patient and describes the patient's cause of hospitalization, whether surgery or treatment was performed, presence of side effects, and others. The first six digits of the DPC code indicate the name of the disease; the DPC codes were used to classify the disease distribution of the sample population into 19 disease groups, and the target group was classified into 28 types of cancer. In the target group, we classified patients based on their status of surgery and extracted all cancer patients who underwent surgery from the database (Figure 1).

Because this study included all cancer patients eligible for participation, sample size calculation was not performed.

All patient selections in this study were based on the DPC database. Therefore, information that was not included in the DPC database, that is, treatment details, stage of disease, and whether or not POM was performed, could not be included in the analysis.

### 2.4 Statistical analysis

Statistical analyses were performed using EZR version 1.35.(Kanda, 2013) For a general understanding of the factors contributing to the development of pneumonia, during hospitalization, age, sex, the incidence of pneumonia during hospitalization, and the category of disease (cancer) were compared between before the introduction of POM (hereinafter referred to as preintroduction) and after the introduction of POM (hereinafter referred to as postintroduction) in the sample population and the target group. Age was compared using the *t*‐test, and sex and the incidence of pneumonia during hospitalization were compared using the chi‐squared test. In the target group, the incidence of pneumonia during hospitalization by age and cancer type were calculated, and the incidence of preintroduction was compared to that of postintroduction using the chi‐squared test. Moreover, multiple logistic regression analysis was performed. The objective variable for the analysis was the onset of pneumonia during hospitalization, and explanatory variables were preintroduction and postintroduction of POM, sex, age, and cancer type. Age was classified into five variables (younger than 50 years, 50–59 years, 60–69 years, 70–79 years, 80 years or older), and cancer type was classified into five variables (brain, stomach, esophagus, lung, and others).

In all analyses, two‐tailed *p* values <.05 were considered statistically significant.

### 2.5 Ethics

This study was conducted in accordance with the ethical guidelines for medical and health research involving human subjects, the ethical guidelines for epidemiological research of the Ministry of Health, Labor and Welfare of Japan, and with approval from the ethics committee of each facility. The details of the study and the method of refusal to participate were publicly available, and written informed consent was not required.

## 3 RESULTS

### 3.1 Characteristics of the patients

Characteristics of the sample population and the target group are summarized in Tables 1 and 2, respectively. In the sample population, significant differences in age and sex were observed preintroduction and postintroduction. There was no significant difference preintroduction and postintroduction in the incidence of pneumonia. In the target group, significant differences were observed preintroduction and postintroduction in age, sex, and incidence of pneumonia.

**Table 1:** Characteristics of the sample population

	Pre‐introduction (*n* = 169,787)	Post‐introduction (*n* = 176,776)	*p* value
Age (year), mean ± *SD*	59.3 ± 22.2	59.5 ± 22.4	.001
Sex (male), n (%)	91,079 (53.6)	93,894 (53.1)	.002
Pneumonia, n (%)	2,682 (1.6)	2,765 (1.6)	.714
Type of disease, n (%)			
Nervous system	12,720 (7.5)	12,450 (7.0)	
Ophthalmic	6,838 (4.0)	6,609 (3.7)	
Otolaryngological	7,945 (4.7)	7,685 (4.3)	
Respiratory	13,834 (8.1)	14,563 (8.2)	
Cardiovascular	19,417 (11.4)	21,078 (11.9)	
Digestive system	40,961 (24.1)	42,224 (23.9)	
Musculoskeletal	7,127 (4.2)	7,474 (4.2)	
Subcutaneous	3,430 (2.0)	4,169 (2.4)	
Breast	2,516 (1.4)	2,960 (1.7)	
Endocrine metabolic	5,649 (3.3)	6,217 (3.5)	
Renal urinary tract and male genital	11,322 (6.6)	11,403 (6.5)	
Female genitalia and puerperal	14,945 (8.8)	15,126 (8.6)	
Blood hematopoiesis	4,331 (2.6)	4,587 (2.6)	
Neonatal	2,091 (1.2)	2,502 (1.4)	
Pediatrics	2,559 (1.5)	2,521 (1.4)	
Trauma, burn, poisoning	10,682 (6.3)	11,518 (6.5)	
Mental illness	490 (0.3)	510 (0.3)	
Others	2,458 (1.4)	2,933 (1.7)	
Unknown	472 (0.3)	247 (0.1)	

*Note.* Pre‐introduction: before the introduction of perioperative oral management. Post‐introduction: after the introduction of perioperative oral management.

**Table 2:** Characteristics of the target group

	Pre‐introduction	Post‐introduction	*p* value
	(*n* = 11,886)	(*n* = 13,668)	
Age (year), mean ± *SD*	65.5 ± 13.5	64.6 ± 13.9	<.001
Sex (male), n (%)	6,385 (53.7)	6,577 (48.1)	<.001
Pneumonia, n (%)	239 (2.0)	114 (0.8)	<.001
Cancer type, n (%)			
stomach	2,060 (17.3)	2,177 (15.9)	
colon	1,493 (12.5)	1,657 (12.1)	
liver and intrahepatic bile duct	1,267 (10.6)	1,234 (9.0)	
rectum and anus	1,118 (9.4)	1,224 (8.9)	
lung	1,002 (8.4)	990 (7.2)	
breast	965 (8.1)	2,010 (14.7)	
cervix and uterine body	917 (7.7)	1,156 (8.4)	
brain	535 (4.5)	526 (3.8)	
esophageal	529 (4.4)	553 (4.0)	
prostate	422 (3.5)	371 (2.7)	
ovary and uterine appendage	258 (2.1)	283 (2.0)	
renal	238 (2.0)	239 (1.7)	
head and neck	220 (1.8)	277 (2.0)	
pancreas	221 (1.8)	215 (1.5)	
non‐melanoma skin	138 (1.1)	141 (1.0)	
thyroid gland	128 (1.0)	183 (1.3)	
gallbladder and extrahepatic bile duct	121 (1.0)	149 (1.0)	
renal pelvis and ureter	94 (0.7)	129 (0.9)	
small intestine and peritoneum	58 (0.4)	66 (0.4)	
melanoma	51 (0.4)	41 (0.2)	
mediastinal	31 (0.2)	30 (0.2)	
soft tissue	16 (0.1)	15 (0.1)	
bone	4 (0.0)	2 (0.0)	
cornea, eye, and appendage	0 (0)	0 (0)	
genital	0 (0)	0 (0)	
vulva	0 (0)	0 (0)	
vagina	0 (0)	0 (0)	
other	0 (0)	0 (0)	

*Note.* Pre‐introduction: before the introduction of perioperative oral management. Post‐introduction: after the introduction of perioperative oral management.

Regarding the disease distribution of the sample population, diseases of the digestive system were the most common preintroduction and postintroduction, with the number of patients increasing in the order of cardiovascular, female genitalia and puerperal, respiratory, and nervous system diseases. Regarding disease distribution in the target group, stomach cancer was the most common, followed by colon, rectum and anus, liver and intrahepatic bile duct, and lung cancer.

### 3.2 Comparison of the number of patients with pneumonia during hospitalization and the incidence of pneumonia preintroduction and postintroduction

The overall population included 169,787 patients who were hospitalized over the 2‐year preintroduction period and approximately 176,776 patients who were hospitalized in the 2‐year postintroduction period. The overall incidence of pneumonia during hospitalization was the same (1.6%) between the preintroduction and postintroduction groups (Table 1).

In the target group, the number of cases of pneumonia preintroduction was 11,886, and the number of cases postintroduction was 13,668. The number of patients with pneumonia during hospitalization was 239 (2.0%) preintroduction and 114 (0.8%) postintroduction, demonstrating a significant difference in the incidence of pneumonia (*p* < .001; Table 2).

### 3.3 Comparison of the incidence of patients with pneumonia by age

**Figure 2:**
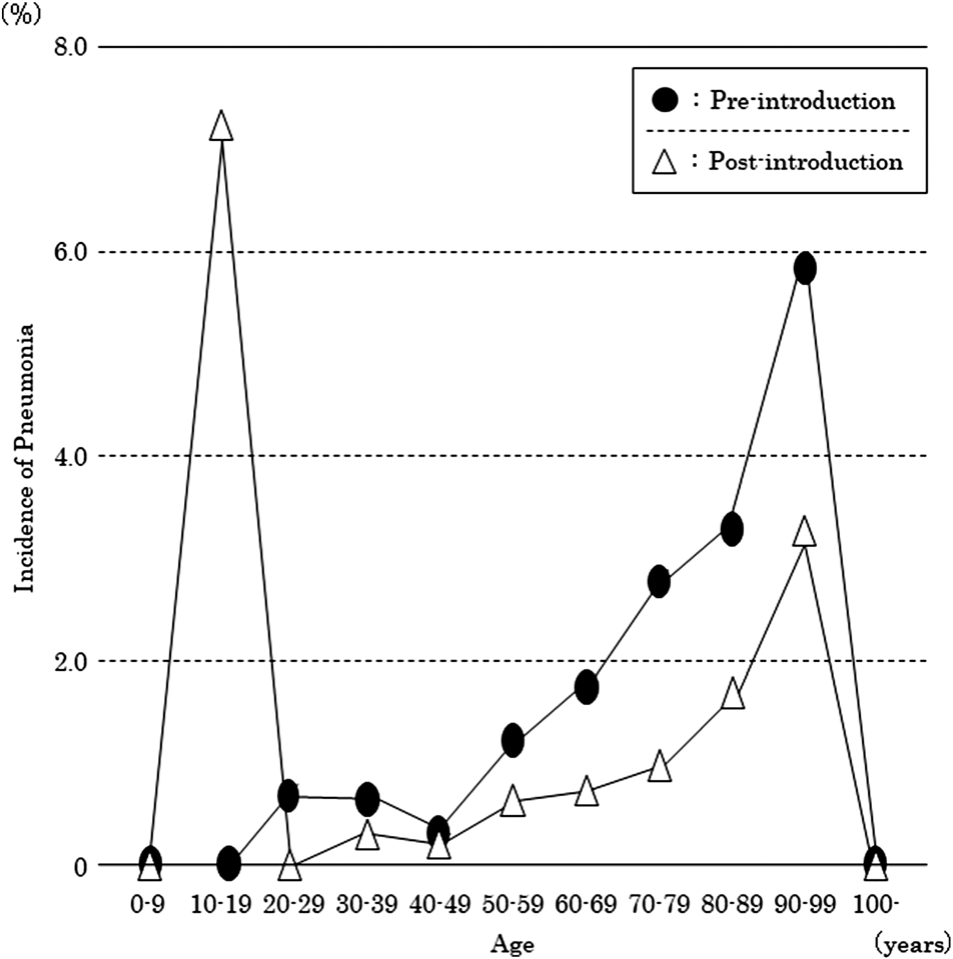
Changes in the incidence of pneumonia by age during hospitalization in cancer patients who underwent surgery (target group). Comparison between before and after the introduction of perioperative oral function management

The incidence of pneumonia in patients aged 10 years was remarkably high (7.1%), amounting to one in every 14 patients developing pneumonia. In the subsequent age groups, however, the incidence was as low as 0–0.3% in patients aged up to 40 years. The incidence increased after 50 years of age, and it was shown that the older the age, the higher the incidence of pneumonia. In a comparison of preintroduction and postintroduction, in the age groups older than 50 years, the incidence of pneumonia was lower postintroduction, and the margin of decrease was largest in 90–99‐year‐old patients (Figure 2)

### 3.4 Differences in the incidence of pneumonia during hospitalization categorized by cancer type and changes preintroduction and postintroduction

Preintroduction, the incidence of pneumonia during hospitalization was highest in the order of stomach, esophageal, small intestine and peritoneal cancer, and brain tumor. Postintroduction, the incidence was highest in the order of brain, small intestine and peritoneal, esophageal, gallbladder, and extrahepatic bile duct tumor.

In a comparison of preintroduction and postintroduction, the incidence of pneumonia during hospitalization decreased in patients with cancers of the gastrointestinal tract, including the stomach, esophagus, and colon, as well as of the lungs. In patients with stomach cancer, the incidence of pneumonia during hospitalization in the preintroduction group was 6.4% (132 of 2,077 cases), whereas the incidence remarkably improved to 1.1% (24 of 2,191 cases) in the postintroduction group. In patients with lung cancer, the incidence of pneumonia during hospitalization was 2.9% (29 of 1,002 cases) preintroduction, but it improved to 1.4% (14 of 990 cases) postintroduction. Similarly, in patients with esophageal cancer, the incidence of pneumonia during hospitalization preintroduction was 4.2% (22 out of 529 cases), and it improved to 2.7% (15 out of 553 cases) postintroduction.

**Figure 3:**
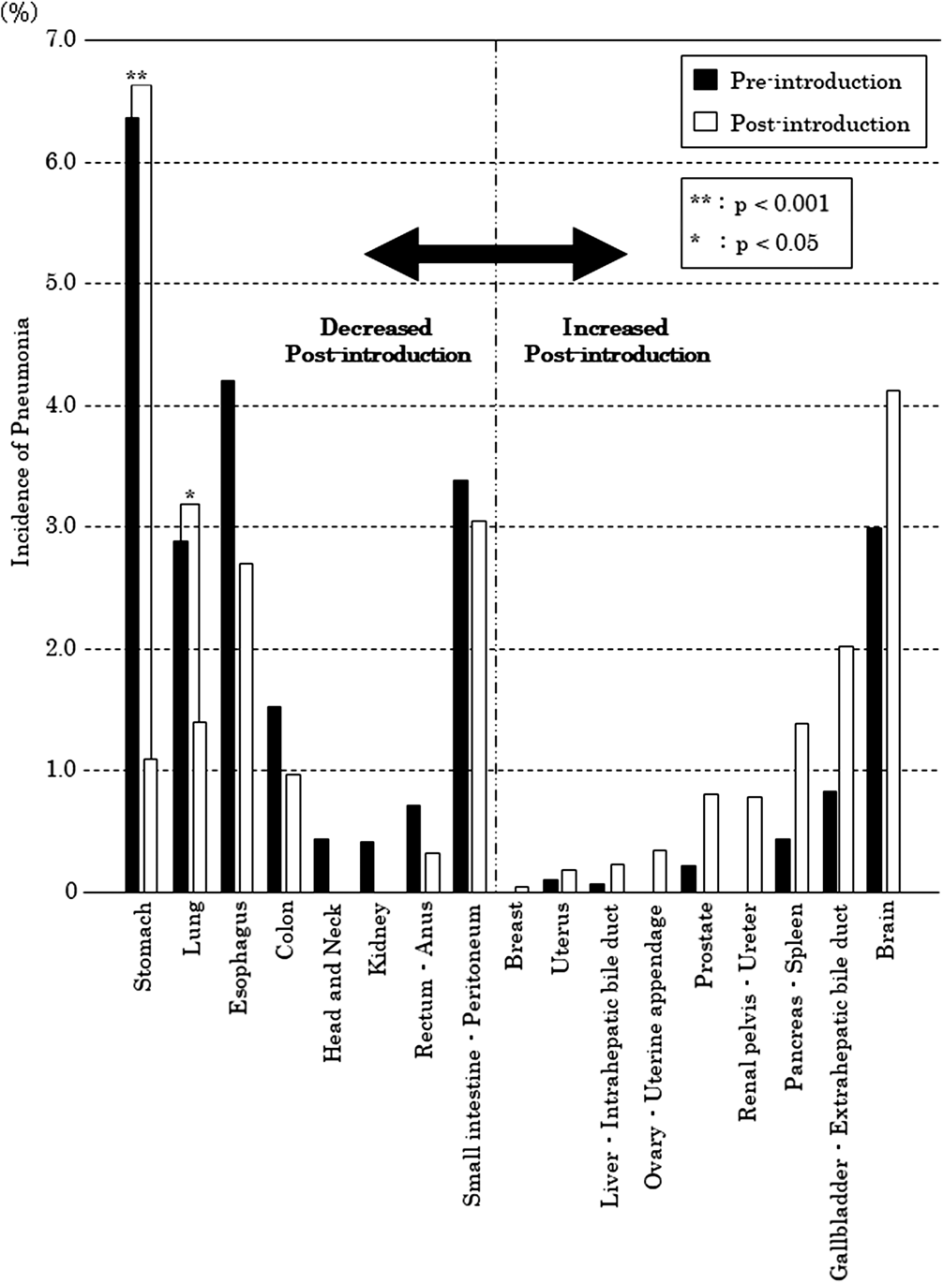
Comparison of the incidence of pneumonia by cancer type. The black bars indicate the period before perioperative oral management (POM) introduction, and the white bars indicate the period after POM introduction. We excluded tumor sites that were not associated with the development of pneumonia in the periods pre‐ and post‐introduction of POM

A statistically significant difference was found in the incidence of stomach (*p* < .001) and lung (*p* < .05) cancers. Prostate cancers and brain tumors showed increase in incidence, but no statistical significance was observed. Besides, the incidence of preintroduction pneumonia was 0.8% in the whole target group, and incidences exceeding this value were noted, in descending order, in patients with tumors at the following sites: the brain, small intestine and peritoneum, esophagus, pancreas and spleen, lung, stomach, and colon (Figure 3).

### 3.5 Factor analysis for pneumonia during hospitalization in the target group

Factors associated with pneumonia were examined by logistic regression analysis with the onset of pneumonia during hospitalization as the objective variable and sex, age, and type of malignant tumor as the explanatory variables.

The risk of pneumonia onset during hospitalization was significantly lower in the postintroduction group than in the preintroduction group, with an odds ratio of 0.44 (95% confidence interval [CI]: 0.35–0.55, *p* < .001).

Sex‐wise, the risk in males was 2.04 times than in females (95% CI: 1.57–2.65, *p* < .001). In the assessment of the odds ratios by age, with age younger than 50 years as the standard, the odds ratio increased with advancing age, showing that patients in their 60s (odds ratio: 2.11, 95% CI: 1.16–3.85, *p* = .014), 70s (odds ratio: 3.03, 95% CI: 1.68–5.46, *p* < .001), and older than 80 years (odds ratio: 4.74, 95% CI: 2.59–8.70, *p* < .001) were at significantly increased risk of developing pneumonia.

Regarding the incidence of pneumonia by cancer type, with cancer type with low incidence of pneumonia as the standard, the odds ratio for cancer types was the highest in patients with brain tumor (odds ratio: 8.95, 95% CI: 6.01–13.30, *p* < .001), followed by stomach cancer (odds ratio: 5.87, 95% CI: 4.44–7.76, *p* < .001), esophageal cancer (odds ratio: 5.49, 95% CI: 3.66–8.23, *p* < .001), and lung cancer (odds ratio: 3.85, 95% CI: 2.64–5.61, *p* < .001; Table 3).

**Table 3:** Risk factors for pneumonia during hospitalization in the target group

	Number of patients	Odds ratio (95% CI)	*p* value	
Introduction of perioperative oral function				
Pre‐introduction	11,886		Standard	
Post‐introduction	13,668	0.44	(0.35–0.55)	<.001
Sex				
Female	12,592		Standard	
Male	12,962	2.04	(1.57–2.65)	<.001
Age (years)				
younger than 50	3,842		Standard	
50–59	3,439	1.80	(0.93−3.49)	.08
60–69	7,297	2.11	(1.16−3.85)	.014
70–79	7,738	3.03	(1.68−5.46)	<.001
80 or older	3,238	4.74	(2.59−8.70)	<.001
Cancer type				
Others	17,121		Standard	
Brain	1,061	8.95	(6.01–13.30)	<.001
Esophagus	1,082	5.49	(3.66–8.23)	<.001
Stomach	4,237	5.87	(4.44–7.76)	<.001
Lung	2,053	3.85	(2.64–5.61)	<.001

Note. Pre‐introduction: before the introduction of perioperative oral management. Post‐introduction: after the introduction of perioperative oral management.

## 4 DISCUSSION

### 4.1 Effectiveness of POM in preventing pneumonia during hospitalization in cancer patients who underwent surgery

This study showed that the overall incidence of pneumonia during hospitalization was 1.6% in both the 2 years preintroduction and postintroduction. In other words, one or two out of every 100 patients developed pneumonia during hospitalization. This finding indicates the need to reduce the burden of pneumonia during hospitalization by analyzing its causative and associated factors and by planning and implementing pneumonia prevention methods. Reported methods of preventing pneumonia include vaccination, oral hygiene, handwashing, avoidance of antibiotics, control of gastroesophageal reflux, and prevention of cerebrovascular disease.(Yamaya, Yanai, Ohrui, Arai, & Sasaki, 2001) Cerebrovascular disease decreases the swallowing and coughing reflexes, increasing the risk of aspiration and, consequently, the risk of developing pneumonia.(Marik & Kaplan, 2003) POM includes swallowing function evaluation and training in addition to oral care. Therefore, it may be possible to prevent pneumonia by improving oral hygiene and swallowing function. Thus, POM is considered an important adjuvant therapy for cancer treatment.

It has been reported that oral care prevents pneumonia.(Akashi, Nanba, Kusumoto, & Komori, 2019; Mizuno et al., 2018; Yoneyama, Yoshida, Matsui, & Sasaki, 1999) Conversely, some treatment guidelines do not require oral care.(Lassen et al., 2009; Mangram, Horan, Pearson, Silver, & Jarvis, 1999) This discrepancy is considered to be due to the fact that there are no high‐level evidence studies on the effects of POM. In order to conduct studies at a high level of evidence, one must first select cases of hospitalized patients at high risk of pneumonia and prescribe POM treatment as an experimental intervention. Such a study would elucidate the triage method for patients at high risk of developing pneumonia and the optimal pneumonia prevention protocol. Thus, it is necessary to establish a method to select hospitalized patients who are at high risk of developing pneumonia. In this study, we examined the incidence of pneumonia in hospitalized patients with various cancer types. The results of this study will help select diseases that may benefit from POM.

In our analysis, the incidence of pneumonia during hospitalization, preintroduction, was 2.0% but decreased to 0.8% postintroduction.

The logistic regression analysis indicated that the odds ratio was approximately 0.4 postintroduction compared with preintroduction and that the risk of pneumonia during hospitalization remarkably decreased.

We targeted cancer patients because they receive POM and, therefore, the effect of POM could be evaluated by comparing the incidence of pneumonia before and after the introduction of POM in this patient sample. Moreover, it is reasonable that we found no difference in the incidence of pneumonia before and after POM introduction in the overall sample population because most did not receive POM.

We consider that POM contributed to the difference in the incidence of pneumonia among cancer patients preintroduction and postintroduction. Although this is only a comparison of the period before and after introduction, as we could not examine whether POM was actually performed or not in this study, POM intervention was considered to be the independent risk factor for developing pneumonia in cancer patients who underwent surgery.

In the future, based on the results of this study, a prospective study will be conducted to evaluate the effectiveness of POM in the specified procedure and frequency.

### 4.2 Comparison of the incidence of pneumonia during hospitalization based on cancer type

In this study, postintroduction, the incidence of pneumonia during hospitalization decreased in patients with cancer of the stomach, lung, esophagus, colon, head and neck, kidney, rectum, anus, and small intestine and peritoneum.

The incidence of respiratory complications after surgery is high in patients with cancer of the esophagus and lung, and the effectiveness of oral care has been reported by retrospective studies.(Akutsu et al., 2010; Iwata et al., 2019; Shin, Kunisawa, Fushimi, & Imanaka, 2019; Tanada, Hoshikawa, Sato, Takahashi, & Koseki, 2019) We validated the findings of previous reports by providing statistical analysis using large‐scale data. Additionally, the present study clarified that the incidence of pneumonia during hospitalization increased in patients with tumors of the pancreas and spleen, kidney, gallbladder, extrahepatic bile duct, and brain postintroduction. Although there are a few reports of pneumonia being the main postoperative complication in these cancer types,(De la Garza‐Ramos et al., 2016; Lonjaret et al., 2017; Nagle et al., 2017) the incidence of pneumonia in brain cancer in particular is high compared to that in other cancer types, necessitating prevention of this complication.

In the logistic regression analysis, patients with brain tumors were at a higher risk of developing pneumonia than were those with malignant esophageal, stomach, and lung tumors. Pneumonia is the main postoperative complication in brain tumor,(Nagle et al., 2017) and in cerebrovascular disorders such as cerebral infarction, it has been reported that the development of pneumonia affects the length of hospitalization and prognosis.(NanZhu, Xin, Xianghua, Jun, & Min, 2019) Conversely, no study has examined the effectiveness of POM in brain tumor patients. Therefore, in the future, more detailed investigations of pneumonia after brain tumor surgery are necessary.

### 4.3 The risk factors for pneumonia during hospitalization

In the target group, the age and sex compositions were different pre‐ and postintroduction. Therefore, multivariate logistic regression analysis was performed for more accurate analysis.

The investigation of related factors showed that age was a related factor because the odds ratio significantly increased after the age of 50 years.

In contrast, as shown in Figure 2, the incidence of pneumonia uniformly and remarkably decreased after the age of 50 years. Therefore, POM appears to effectively prevent pneumonia in patients older than 50 years.

Age could be considered an indicator to triage cancer patients with a high risk of developing pneumonia. For example, if patients older than 70 years are prioritized, the number of target patients decreases to approximately 1/3, and in this manner, a greater number of high‐risk patients could be treated.

POM is only performed upon request by the patient's attending physician in Japan, and it may be challenging for the attending physician to determine which patients require POM. Therefore, age may be a readily accessible POM indicator.

The incidence of hospital‐acquired pneumonia increases after the age of 60 years, and the rate of aspiration pneumonia has been reported to be 50% in patients in their 60s, 70% in patients in their 70s, and over 80% in patients older than 80 years.(Teramoto et al., 2008) Our data showed a trend similar to that reported by a previous study; it is consider that most cancer patients develop aspiration pneumonia after surgery. As a method of preventing aspiration pneumonia, oral care, including evaluation and training of swallowing function, brushing, and removal of teeth that that are poorly maintained, is recommended.(Mandell & Niederman, 2019) Therefore, active intervention by POM including evaluation and training of swallowing function is considered necessary for people older than 60 years.

Sex‐wise, the risk of pneumonia development was almost twice as high (*p* < .001) in males. It has been reported that males are at a higher risk of developing pneumonia requiring hospitalization, and older men have a higher risk of developing aspiration pneumonia due to reduced swallowing function.(Lin et al., 2019) Furthermore, swallowing function training is known to effectively prevent pneumonia.(Miki et al., 2018) Therefore, it is necessary to perform POM not only for oral care but also for evaluation and training of swallowing function for the prevention of pneumonia in elderly man.

## 5 LIMITATIONS OF THIS STUDY

This study has some research limitations. As we only examined data included in the DPC database, information that was not provided, that is, treatment details, stage of disease, and whether or not POM was performed, could not be included in this study. Thus, individual cases could not be analyzed in detail. Therefore, it was difficult to examine the relationship between the implementation of POM and the degree of progression of the original disease, the magnitude of surgical invasion, postoperative course, incidence of pneumonia during hospitalization, and other variables. In order to clarify these points, it is necessary to subject the data of the high‐risk groups to more detailed analysis that will account for background factors. The present study provides preliminary data for prospective studies. Because some important background factors were validated through this study, we plan to examine the pneumonia‐preventing effect of POM after controlling for these factors in the future.

## 6 CONCLUSION

In cancer patients who had undergone surgery, the incidence of pneumonia significantly decreased after the introduction of POM, and males were twice as likely to develop pneumonia. Patients older than 60 years had a significantly higher risk of developing pneumonia than had patients younger than 50 years. Finally, patients with cancer of the brain, esophagus, stomach, and lung were at higher risk of developing pneumonia than were patients with other types of cancer.

## CONFLICT OF INTEREST

All authors declare no conflicts of interest associated with this manuscript.
